# Supplementary material for: HIVprotI: an integrated web based platform for prediction and design of HIV proteins inhibitors
Source: J Cheminform. 2018 Mar 9;10:12. doi: 10.1186/s13321-018-0266-y (PMC5845081; doi:10.1186/s13321-018-0266-y)
Supplement: Supplementary file 2 — Additional file 2. Source code of HIVProtI web server. [file 13321_2018_266_MOESM2_ESM.zip › HIVprotI_Source-code/hivprotI/weka-3-6-6/doc/weka/core/matrix/QRDecomposition.html]

QRDecomposition


---


|  |  |  |
| --- | --- | --- |
| **Overview** \| **Package** \| **Class** \| **Tree** \| **Deprecated** \| **Index** \| **Help** \| **Weka's home** | |  |
| **PREV CLASS**   **NEXT CLASS** | **FRAMES**    **NO FRAMES**     **All Classes** |  |
| SUMMARY: NESTED \| FIELD \| CONSTR \| METHOD | DETAIL: FIELD \| CONSTR \| METHOD |  |


---


## weka.core.matrix Class QRDecomposition

```
java.lang.Object
  weka.core.matrix.QRDecomposition
```

**All Implemented Interfaces:**: java.io.Serializable, RevisionHandler

---

``` public class QRDecomposition extends java.lang.Object implements java.io.Serializable, RevisionHandler ```

QR Decomposition.

For an m-by-n matrix A with m >= n, the QR decomposition is an m-by-n
orthogonal matrix Q and an n-by-n upper triangular matrix R so that A = Q\*R.

The QR decompostion always exists, even if the matrix does not have full
rank, so the constructor will never fail. The primary use of the QR
decomposition is in the least squares solution of nonsquare systems of
simultaneous linear equations. This will fail if isFullRank() returns false.

Adapted from the JAMA package.

**Version:**
:   $Revision: 1.4 $

**Author:**
:   The Mathworks and NIST, Fracpete (fracpete at waikato dot ac dot nz)

**See Also:**: Serialized Form

---

**Constructor Summary**

`QRDecomposition(Matrix A)`
  
          QR Decomposition, computed by Householder reflections.


| **Method Summary** | |
| --- | --- |
| `Matrix` | `getH()`             Return the Householder vectors |
| `Matrix` | `getQ()`             Generate and return the (economy-sized) orthogonal factor |
| `Matrix` | `getR()`             Return the upper triangular factor |
| `java.lang.String` | `getRevision()`             Returns the revision string. |
| `boolean` | `isFullRank()`             Is the matrix full rank? |
| `Matrix` | `solve(Matrix B)`             Least squares solution of A\*X = B |

**Methods inherited from class java.lang.Object**

`equals, getClass, hashCode, notify, notifyAll, toString, wait, wait, wait`

**Constructor Detail**

### QRDecomposition

```
public QRDecomposition(Matrix A)
```

:   QR Decomposition, computed by Householder reflections.

    **Parameters:**: `A` - Rectangular matrix


**Method Detail**

### isFullRank

```
public boolean isFullRank()
```

:   Is the matrix full rank?

    :   **Returns:**: true if R, and hence A, has full rank.

---


### getH

```
public Matrix getH()
```

:   Return the Householder vectors

    :   **Returns:**: Lower trapezoidal matrix whose columns define the reflections

---


### getR

```
public Matrix getR()
```

:   Return the upper triangular factor

    :   **Returns:**: R

---


### getQ

```
public Matrix getQ()
```

:   Generate and return the (economy-sized) orthogonal factor

    :   **Returns:**: Q

---


### solve

```
public Matrix solve(Matrix B)
```

:   Least squares solution of A\*X = B

    :   **Parameters:**: `B` - A Matrix with as many rows as A and any number of columns. **Returns:**: X that minimizes the two norm of Q\*R\*X-B. **Throws:**: `java.lang.IllegalArgumentException` - Matrix row dimensions must agree.: `java.lang.RuntimeException` - Matrix is rank deficient.

---


### getRevision

```
public java.lang.String getRevision()
```

:   Returns the revision string.

    :   **Specified by:**: `getRevision` in interface `RevisionHandler`
    :   **Returns:**: the revision


---


|  |  |  |
| --- | --- | --- |
| **Overview** \| **Package** \| **Class** \| **Tree** \| **Deprecated** \| **Index** \| **Help** \| **Weka's home** | |  |
| **PREV CLASS**   **NEXT CLASS** | **FRAMES**    **NO FRAMES**     **All Classes** |  |
| SUMMARY: NESTED \| FIELD \| CONSTR \| METHOD | DETAIL: FIELD \| CONSTR \| METHOD |  |


---
